# Optimal Duration of Adjuvant Platinum–Etoposide in High-Risk Merkel Cell Carcinoma

**DOI:** 10.3390/medicina62050882

**Published:** 2026-05-04

**Authors:** Ronen Brenner, Hanna T. Frumin Edri, Keren Rouvinov, Noa Shani Shrem, Amichay Meirovitz, Sabri El-Saied, Ilia Berezhnov, Anna Ievko, Sofiia Turaieva, Shlomit Fenig, Nashat Abu Yasin, Eyal Fenig, Samer Hussany, Alexander Yakobson, Walid Shalata

**Affiliations:** 1 Oncology Institute, Edith Wolfson Medical Center, Holon 58220, Israel; 2 Faculty of Medicine, Tel Aviv University, Tel Aviv 69978, Israel; 3 The Legacy Heritage Cancer Center, Dr. Larry Norton Institute, Soroka Medical Center, Beer Sheva 84105, Israel; 4 Faculty of Health Sciences, Ben Gurion University of the Negev, Beer Sheva 84105, Israel; 5 Institute of Oncology, Kaplan Medical Center, Faculty of Medicine, Hebrew University, Jerusalem 91905, Israel; 6 Institute of Oncology, Davidoff Center, Rabin Medical Center, Beilinson Hospital, Petah Tikva 49414, Israel

**Keywords:** Merkel cell carcinoma, adjuvant chemotherapy, platinum–etoposide, radiotherapy, disease-free survival, overall survival, neuroendocrine carcinoma

## Abstract

*Background and Objectives:* Merkel cell carcinoma (MCC) is a rare and aggressive neuroendocrine skin malignancy associated with high rates of recurrence and disease-specific mortality. Although adjuvant platinum–etoposide chemotherapy is used in high-risk disease, the optimal number of treatment cycles has not been established. *Materials and Methods*: This multicenter retrospective cohort study included 104 patients with resected high-risk MCC (pathological stage IIB–III) treated at Israeli medical centers between September 1985 and February 2021. Patients were assigned to one of three treatment groups: radiotherapy alone, four cycles of platinum–etoposide plus radiotherapy, or six cycles of platinum–etoposide plus radiotherapy. The chemotherapy regimen consisted of cisplatin or carboplatin combined with etoposide in 21-day cycles, with the first two cycles administered concurrently with radiotherapy. Primary endpoints were disease-free survival (DFS) and overall survival (OS), analyzed using the Kaplan–Meier method and multivariable Cox proportional hazards regression. *Results*: Four cycles of adjuvant platinum–etoposide combined with radiotherapy were associated with the most favorable survival outcomes at all follow-up time points. Five-year DFS and OS in the four-cycle group were 65% (95% CI: 58–72%) and 75% (95% CI: 68–82%), respectively, compared with 55% and 60% in the six-cycle group, and 40% and 45% in the radiotherapy-only group (*p* < 0.001). The survival advantage of four cycles over radiotherapy alone was sustained at 10- and 20-year follow-up (*p* < 0.0001). In patients with stage III disease and nodal involvement, the four-cycle group achieved a median DFS of 93 months and a median OS of approximately 110 months, significantly exceeding outcomes in both the six-cycle and radiotherapy-alone groups. No statistically significant survival benefit from chemotherapy was identified in the small subgroup of patients with stage IIB/T4N0 disease. *Conclusions*: In patients with high-risk resected MCC, the addition of adjuvant platinum–etoposide chemotherapy to radiotherapy significantly improves DFS and OS, with the greatest benefit observed in patients with stage III disease and lymph node involvement. Four cycles represent an optimal treatment duration, delivering durable long-term survival benefit without the need for more prolonged chemotherapy exposure. These findings support a risk-adapted multimodality approach and provide real-world evidence to guide adjuvant therapy decisions in this rare and aggressive malignancy.

## 1. Introduction

Merkel cell carcinoma (MCC) is a rare and highly aggressive neuroendocrine malignancy of the skin characterized by a high risk of recurrence and disease-specific mortality. The incidence of MCC has increased steadily over recent decades, largely attributed to an aging population, increased ultraviolet (UV) exposure, and improved diagnostic recognition [1,2]. Two major pathogenic pathways have been identified in MCC: UV-induced carcinogenesis and integration of the Merkel cell polyomavirus (MCPyV), which is detected in the majority of tumors in many geographic regions [3]. Despite biological differences between virus-positive and UV-driven tumors, both subtypes are associated with aggressive clinical behavior and a substantial risk of regional and distant metastasis [4].

The standard management of localized MCC involves wide local excision with appropriate staging of regional lymph nodes, frequently combined with adjuvant radiotherapy to improve local and regional control [5]. Multiple retrospective studies have demonstrated that adjuvant radiotherapy is associated with reduced locoregional recurrence and improved disease control, particularly in patients with high-risk features such as positive lymph nodes, large tumors, or close surgical margins [6]. However, the role of systemic therapy in the adjuvant setting remains controversial.

Historically, cytotoxic chemotherapy regimens used in MCC have been extrapolated from treatment paradigms for small-cell lung cancer because of the shared neuroendocrine phenotype and chemosensitivity of these malignancies. The most commonly utilized regimen consists of a platinum compound, such as cisplatin or carboplatin, combined with etoposide (VP-16). Although these regimens demonstrate relatively high initial response rates in metastatic MCC, responses are typically short-lived, and relapses occur frequently [7].

Several retrospective studies and large database analyses have evaluated the benefit of adjuvant chemotherapy in patients with localized or node-positive MCC; however, results have been inconsistent. Most available evidence suggests that adjuvant chemotherapy does not significantly improve overall survival and may be associated with considerable toxicity, particularly in the predominantly elderly MCC population, and only subgroups may gain a benefit [8,9,10]. Consequently, current clinical guidelines generally do not recommend routine use of adjuvant chemotherapy outside of selected high-risk cases [11].

In recent years, the therapeutic landscape of advanced MCC has changed significantly with the introduction of immune checkpoint inhibitors targeting the PD-1/PD-L1 pathway. Agents such as Avelumab and Pembrolizumab have demonstrated durable responses and improved survival outcomes in patients with metastatic disease, leading to their adoption as standard first-line systemic therapy in advanced MCC [12,13]. Nevertheless, the optimal role of systemic therapy in earlier disease stages remains unclear. Among patients who do receive adjuvant chemotherapy, the optimal duration of treatment has not been established. In clinical practice, platinum–etoposide regimens are commonly administered for four to six cycles, largely extrapolated from small-cell lung cancer treatment protocols. However, there is a lack of evidence evaluating whether additional cycles provide meaningful clinical benefit in MCC [7]. Data comparing outcomes between patients treated with shorter versus longer chemotherapy courses are limited, and the potential balance between treatment efficacy and treatment-related toxicity remains poorly defined.

Given the rarity of MCC and the absence of prospective randomized trials addressing chemotherapy duration, retrospective real-world analyses may provide valuable insights into treatment optimization. Therefore, the aim of this study is to evaluate outcomes in patients with MCC with high risk of recurrency (patients with MCC with high risk of recurrence, pathological T4 without lymph node (LN) involvement (stage IIB), and any T with LN involvement (stage III)) that were treated with adjuvant platinum–etoposide chemotherapy, with particular focus on comparing clinical outcomes between patients receiving four versus six cycles of treatment. By examining survival outcomes and recurrence patterns associated with different chemotherapy durations, this study seeks to contribute to the limited evidence base guiding systemic adjuvant therapy in MCC.

## 2. Materials and Methods

### 2.1. Study Design and Patient Population

This multicenter retrospective cohort study included patients diagnosed with localized or locally advanced MCC who underwent surgical resection followed by adjuvant treatment with either radiotherapy alone or radiotherapy combined with chemotherapy. Eligible patients were those with high-risk pathological features, defined as pathological T4 tumors without lymph node involvement (stage IIB) or tumors of any T stage with lymph node involvement. Patients treated between September 1985 and February 2021 were identified from medical centers in Israel.

Patients were categorized into three groups based on adjuvant treatment received:

1. Patients who received six cycles of adjuvant platinum–etoposide chemotherapy plus radiotherapy.
2. Patients who received four cycles of adjuvant platinum–etoposide chemotherapy plus radiotherapy.
3. Patients who received radiotherapy alone, without chemotherapy.

### 2.2. Data Collection

Eligible patients were identified using pathology databases and oncology department registries, applying standardized diagnostic codes. Clinical and pathological data were extracted from electronic medical records and entered into a predefined data collection template by trained investigators. Variables collected included:

Demographics: age at diagnosis, sex.

Tumor characteristics: location, stage according to TNM classification.

Treatment information: surgery, radiation dose, radiotherapy technique, and number of chemotherapy cycles.

Disease stage at diagnosis was determined using the American Joint Committee on Cancer (AJCC) TNM classification, incorporating the extent of the primary tumor (T), regional lymph node involvement (N), and presence or absence of distant metastases (M). Mortality data were obtained from the Israel Ministry of Interior national registry, with the last follow-up conducted through May 2025.

### 2.3. Inclusion Criteria

Patients were eligible if they

Were ≥18 years of age at diagnosis;

Had histopathologically confirmed MCC with any T stage, N stage, and no distant metastases (M0);

Received radiotherapy, with or without chemotherapy (four or six cycles), as part of initial treatment;

Had complete follow-up information available from one of the participating centers.

### 2.4. Exclusion Criteria

Patients were excluded if they

Had no lymph node involvement or pathologically T was less than 4, or if they had distant metastases at diagnosis;

Received prior radiotherapy or systemic therapy for MCC;

Had a diagnosis other than MCC;

Lacked sufficient follow-up to determine disease-free survival (DFS) or overall survival (OS).

### 2.5. Treatment Administration

Patients received multimodal treatment including surgery, radiotherapy, and systemic chemotherapy when indicated. The chemotherapy regimen consisted of cisplatin (20 mg/m^2^, days 1–5) or cisplatin (75 mg/m^2^, days 1) or carboplatin (AUC5) and etoposide (100 mg/m^2^, days 1, 2, 3) in 21-day cycles, administered for four or six cycles. The first two cycles were given concurrently with radiotherapy.

Radiotherapy was delivered to the primary tumor and regional lymphatic drainage areas using 3D conformal radiotherapy (3D-CRT) or intensity-modulated radiotherapy (IMRT), with total doses ranging from 45 to 50 Gy in 25 fractions, plus a sequential boost of 9–10 Gy to areas of macroscopic disease when present.

### 2.6. Statistical Analysis

Categorical variables were summarized as frequencies and percentages. Continuous variables were assessed for normality and summarized as means ± SD or medians with interquartile ranges (IQR). Comparisons between groups were performed using

Continuous variables: One-way ANOVA or Kruskal–Wallis test, as appropriate;

Categorical variables: Chi-square or Fisher’s exact test.

Survival outcomes (OS and DFS) were analyzed using the Kaplan–Meier method, with differences between groups assessed via the log-rank test. Multivariable Cox proportional hazards models were used to examine the impact of chemotherapy administration on survival while adjusting for confounders (age, sex, stage, tumor location, and sun exposure). *p*-values <0.05 were considered statistically significant. Analyses were performed using IBM SPSS (version 29) Statistics (IBM Corp., Armonk, NY, USA).

In addetion, we used the AI tools Claude 4 and ChatGPT Deep Research version 5.3 for the purposes of assisting in identifying grammatical issues.

## 3. Results

A total of 104 patients were included in the analysis, with a mean age at diagnosis of 70.6 ± 14.0 years. Patients treated with radiotherapy alone were generally older (76.5 ± 13.6 years) compared with those receiving six cycles of chemotherapy (72.3 ± 11.7 years) or four cycles (68.1 ± 14.3 years). Overall, 61 patients (59%) were male, and 43 (41%) were female. The proportion of males was higher in the six-cycle group (71%) and four-cycle group (62%), whereas females were more represented in the radiotherapy-only group (56%). Regarding disease stage, stage IIIB was the most common, accounting for 60 patients (58%), followed by stage IIIA in 32 patients (31%) and stage IIB in 12 patients (12%). Stage IIIB disease predominated among patients receiving chemotherapy, particularly in the four-cycle group (71%) and six-cycle group (64%), while patients treated with radiotherapy alone more frequently had stage IIIA disease (56%). Physicians demonstrated a clear preference for radiotherapy-only protocols in the elderly population, with this group maintaining the highest mean age (76.5 + 13.6 years) compared to the younger cohorts receiving four-cycle (68.1 + 14.3 years) or six-cycle (72.3 + 11.7 years) chemotherapy regimens. This trend indicates a clinical prioritization of local control over systemic toxicity in geriatric patients. Furthermore, the intensity of treatment appeared to correlate with disease severity; the four-cycle chemotherapy group represented the most advanced disease state, with 71% of patients presenting as Stage IIIB, whereas the radiotherapy-only group was predominantly Stage IIIA (56%). Interestingly, anatomical site also appeared to influence the decision-making process, as 29% of patients in the four-cycle chemotherapy group presented with groin-located tumors, a presentation entirely absent (0%) in the radiotherapy-only cohort. Collectively, these data suggest that while chemotherapy was the preferred systemic intervention for younger patients with advanced nodal involvement (Stage IIIB), physicians opted for a de-escalated, local-only approach for older patients or those with lower-stage (IIIA) disease to balance therapeutic efficacy with physiological resilience (Table 1).

The median DFS was longest in the four-cycle group, which was 42 months, followed by the 6-cycle group, which was 38 months, and shortest in the radiotherapy-only group, which was 24 months.

**Figure 1:**
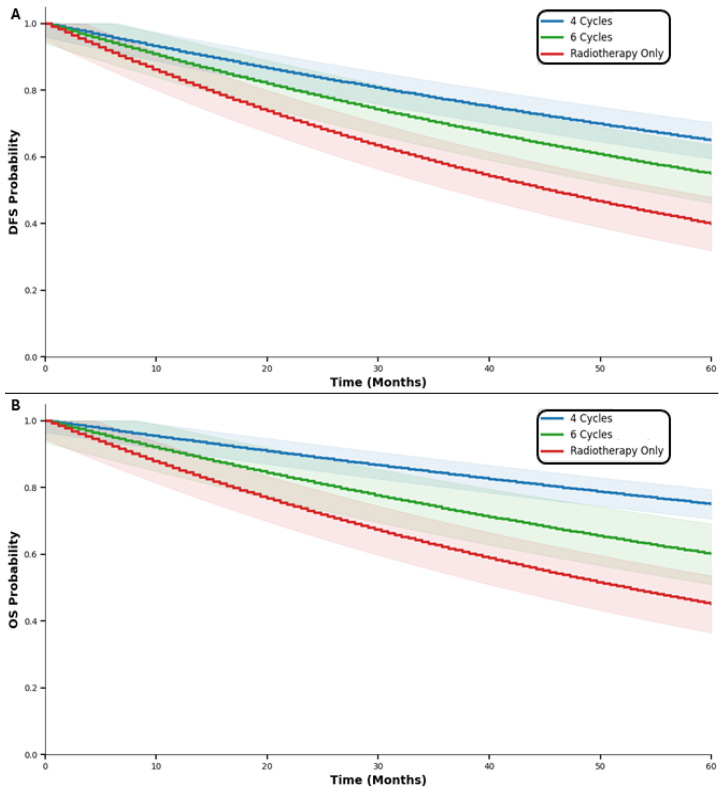
The 5-year outcomes of the patients by disease-free survival (**A**) and OS (**B**) according to the type of treatment.

**Figure 2:**
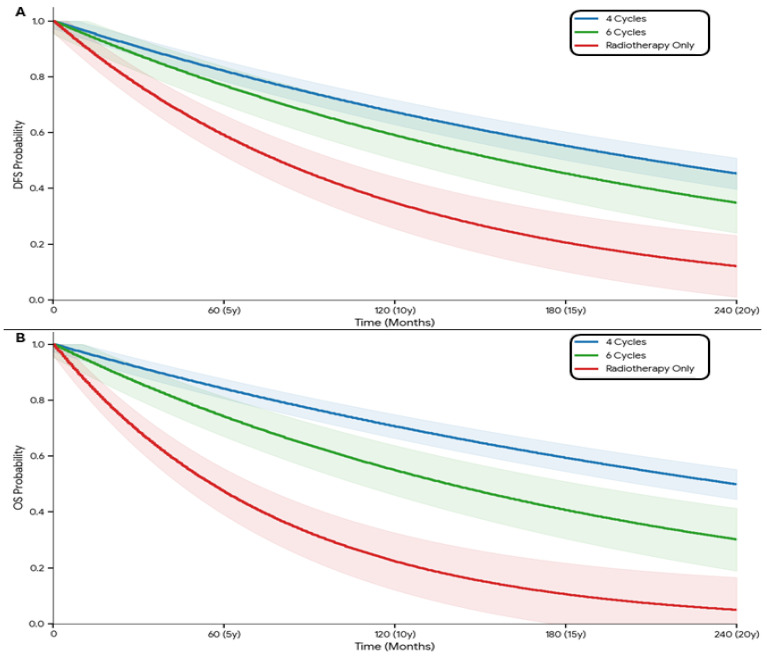
The 20-year outcomes of the patients with disease-free survival (**A**) and OS (**B**) according to the type of treatment.

Overall, when comparing the outcomes of the patient by the DFS and OS results, in comparison to the type of treatment administered, it was shown that the survival outcomes differed significantly between treatment groups. For DFS, the four-cycle group had the highest estimated survival at 5 years (65%, 95% CI: 58–72%), followed by the six-cycle group (55%, 95% CI: 43–67%) and the radiotherapy-only group (40%, 95% CI: 32–48%), with differences remaining significant at 10 and 20 years (*p* < 0.001 and *p* < 0.0001, respectively). Similarly, overall survival (OS) was superior in the four-cycle group across all time points, reaching 75% (95% CI: 68–82%) at 5 years, compared with 60% (95% CI: 47–73%) for six-cycle and 45% (95% CI: 34–56%) for RT only (*p* < 0.001), (Table 2, Figure 1 and Figure 2).

The comparison of four- and six-cycle chemotherapy versus radiotherapy alone, stratified by sex, did not reach statistical significance. For DFS, the *p*-value was 0.251, and for OS, the *p*-value was 0.187, indicating no significant difference between males and females in these treatment groups.

For patients with stage II disease with pathological T4 tumors (only 12 patients), there were no significant differences in survival outcomes between treatment strategies. Median DFS and OS were comparable between radiotherapy alone and radiotherapy combined with 4 or 6 cycles of chemotherapy, with *p*-values of 0.32 and 0.28, respectively.

**Figure 3:**
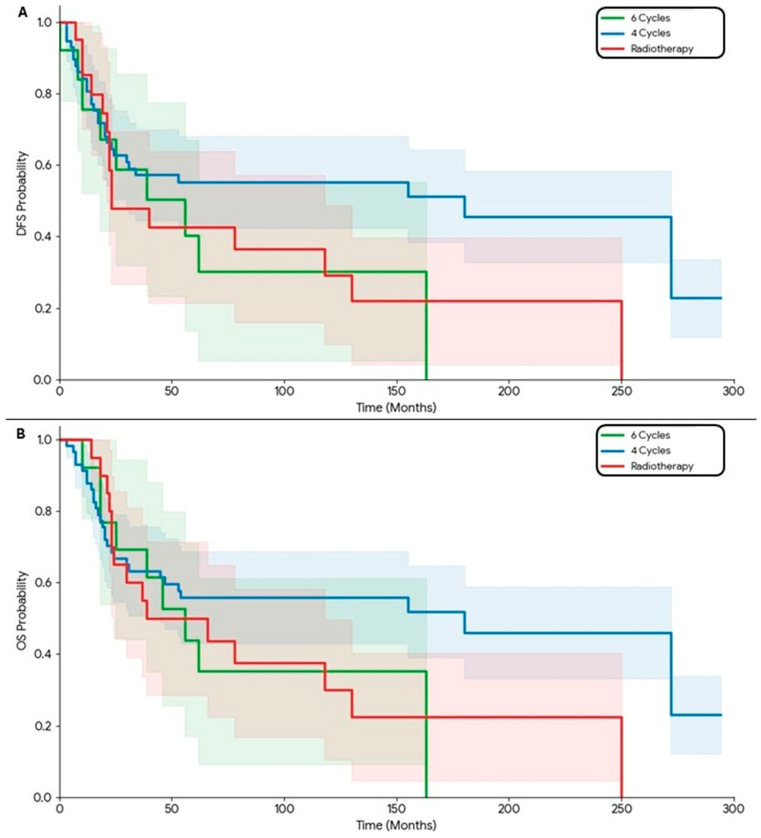
Stage 3, (**A**) the disease-free survival curve demonstrates the effectiveness of systemic therapy in preventing recurrence. Patients receiving 4 cycles of chemotherapy (blue line) maintained a significantly higher proportion of disease-free status over time compared with radiotherapy alone (*p* < 0.05). (**B**) The overall survival curve (**B**) mirrors this trend, indicating that the 4-cycle chemotherapy regimen provides superior overall survival outcomes for Stage III patients. In contrast, the radiotherapy group shows a steeper decline, highlighting that local treatment alone is often insufficient to address the high systemic risk associated with nodal involvement (*p* < 0.05).

In patients at the highest risk of recurrence—stage III disease with lymph node involvement—systemic control via chemotherapy proved critical. The dataset includes a substantial number of patients in the four-cycle chemotherapy group, providing a robust basis for statistical analysis. Analysis of DFS across treatment groups revealed that patients receiving four cycles of chemotherapy experienced the longest disease-free intervals, with a median DFS of 93 months, demonstrating a sustained long-term plateau. Patients treated with six cycles had a median DFS of 42 months, although the small sample size limits interpretability. In contrast, patients receiving radiotherapy alone had the shortest DFS, with a median of 23 months, highlighting the reduced effectiveness of local-only therapy in preventing recurrence (Figure 3A). The OS analysis followed a similar trend. Patients in the four-cycle chemotherapy group achieved the longest overall survival, with a median OS of approximately 110 months, indicating robust long-term benefit. The six-cycle group showed a median OS of 56 months, reflecting both the smaller cohort and reduced systemic exposure compared with the four-cycle group. Patients receiving radiotherapy alone had the lowest OS, with a median of 34 months, consistent with the higher risk of systemic failure in the absence of chemotherapy (Figure 3B).

**Figure 4:**
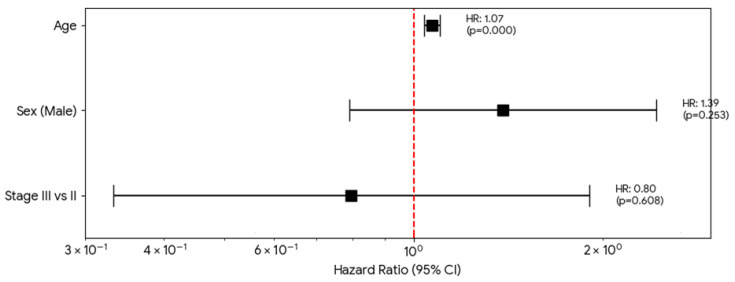
Cox proportional hazards model. This analysis adjusts for age, TNM stage, and sex to determine whether there are differences in survival between the treatment groups (radiotherapy, 4 cycles, and 6 cycles).

In the multivariate Cox proportional hazards analysis, after adjusting for potential confounders, age at diagnosis was the only independent predictor of overall survival. Each additional year of age was associated with a 7% increase in the hazard of death (HR 1.07; 95% CI 1.04–1.10; *p* < 0.001). Once this age-related risk was accounted for, no statistically significant difference in survival was observed between the treatment arms. Notably, neither biological sex (*p* = 0.253) nor clinical stage (*p* = 0.608) demonstrated a significant independent impact on survival. These results imply that while age is the dominant prognostic factor, the risk profile across Stages IIB, IIIA, and IIIB remains consistently hazardous, reinforcing the clinical necessity for adjuvant therapy regardless of specific sub-stage or treatment cycle duration (Figure 4).

## 4. Discussion

The standard of care for localized MCC consists of wide local excision paired with regional lymph node staging. Adjuvant radiotherapy is frequently utilized to enhance locoregional control, with proven efficacy in reducing recurrence among high-risk patients. However, the role of adjuvant systemic therapy remains a subject of clinical debate. In our cohort of 104 patients, survival outcomes differed significantly between treatment groups. Patients receiving four cycles of chemotherapy achieved the highest long-term DFS and OS, with 5-year survival rates of 65% and 75%, respectively. In contrast, radiotherapy alone was associated with inferior outcomes, with 5-year DFS and OS rates of 40% and 45%. These findings suggest that systemic therapy contributes meaningfully to disease control in MCC, particularly in patients with nodal involvement. Importantly, the survival advantage associated with chemotherapy persisted at long-term follow-up, including 10- and 20-year analyses.

Our findings are consistent with previous reports indicating that adjuvant radiotherapy improves local control in MCC but may be insufficient for preventing systemic relapse. Several retrospective analyses have demonstrated that MCC frequently disseminates early, particularly in node-positive disease, highlighting the potential importance of systemic therapy in high-risk populations [14,15]. Similarly, analyses from large national databases have suggested that patients with nodal involvement may derive benefit from multimodality treatment approaches combining surgery, radiotherapy, and systemic therapy [16].

Despite this rationale, the role of adjuvant chemotherapy in MCC remains controversial. Earlier studies reported limited survival benefit and considerable toxicity associated with chemotherapy regimens derived from small-cell lung cancer protocols, such as platinum plus etoposide [17,18,19]. Consequently, contemporary clinical guidelines generally do not recommend routine use of adjuvant chemotherapy outside selected high-risk cases [20]. However, many of these studies were conducted before the widespread use of modern staging techniques and improved radiotherapy approaches, which may influence recurrence patterns and survival outcomes.

Our data suggest that systemic therapy may indeed provide meaningful benefit in selected populations, particularly patients with stage III disease. In the subgroup of patients with nodal involvement, four cycles of chemotherapy were associated with a significant improvement in both DFS and OS compared with radiotherapy alone. These findings support the hypothesis that systemic micro-metastatic disease contributes to recurrence in stage III MCC and that chemotherapy may help eradicate occult tumor cells.

Interestingly, increasing chemotherapy intensity beyond four cycles did not translate into improved outcomes. Patients receiving six cycles demonstrated intermediate survival results that were inferior to those observed in the four-cycle group. Several explanations may account for this observation. First, the six-cycle cohort was relatively small (n = 14), limiting statistical power. Second, treatment selection bias may have influenced these results, as patients receiving more intensive therapy may have had more aggressive disease characteristics. Finally, prolonged chemotherapy exposure may increase toxicity without improving systemic tumor control.

These findings raise an important clinical question regarding the optimal duration of adjuvant chemotherapy in MCC. Based on our results, four cycles appear sufficient to achieve maximal therapeutic benefit while minimizing unnecessary treatment exposure. This concept parallels findings from other malignancies in which shorter chemotherapy durations have demonstrated similar efficacy with reduced toxicity [21,22].

Another important observation from our study is the influence of patient characteristics on treatment selection. Physicians tended to prescribe radiotherapy alone for older patients, likely reflecting concerns regarding chemotherapy tolerability in this population. Indeed, the radiotherapy-only group had the highest mean age in our cohort. This pattern reflects real-world clinical decision-making, where treatment strategies must balance oncologic benefit with patient frailty and comorbidities.

Additionally, tumor stage played a major role in determining treatment intensity. Patients with stage IIIB disease were more likely to receive systemic chemotherapy, whereas those with stage IIIA or IIB disease were more often treated with radiotherapy alone. This risk-adapted strategy aligns with current treatment principles for MCC, where therapy intensity is tailored to recurrence risk and disease burden.

In contrast, chemotherapy did not appear to provide a clear survival benefit in the subgroup of patients with stage II disease and pathological T4 tumors. In this small subset, DFS and OS were comparable between radiotherapy alone and combined chemoradiotherapy. This observation suggests that systemic therapy may be less critical in patients without nodal involvement, although the small sample size limits definitive conclusions.

The present study has several limitations. First, the retrospective and non-randomized design introduces potential selection bias in treatment allocation. In particular, patients treated with radiotherapy alone were generally older, whereas patients receiving chemotherapy tended to have more advanced disease. These baseline imbalances may have influenced survival outcomes and limited causal interpretation of treatment effects.

Second, although multivariable Cox regression analysis was performed to adjust for potential confounders such as age, stage, and sex, residual confounding cannot be excluded due to the retrospective nature of the dataset and the limited sample size.

Third, the relatively small number of patients in the six-cycle chemotherapy group limits statistical power and restricts the ability to draw definitive conclusions regarding the optimal duration of treatment.

Fourth, the long inclusion period spanning multiple decades introduces potential temporal heterogeneity. During this time, significant advances occurred in diagnostic imaging, staging accuracy, radiotherapy techniques, surgical management, and supportive care, all of which may have influenced patient outcomes. Although exploratory analyses did not demonstrate clear differences between treatment eras, these findings should be interpreted cautiously due to limited statistical power.

Fifth, the study period largely precedes the widespread adoption of immune checkpoint inhibitors, which have recently emerged as highly effective therapies for advanced Merkel cell carcinoma [23,24,25]. Therefore, the applicability of these findings to contemporary treatment paradigms incorporating immunotherapy remains uncertain. Future studies should evaluate the role of chemotherapy in the context of modern immunotherapy-based treatment strategies.

Despite these limitations, our study provides real-world evidence regarding the role of chemotherapy in the adjuvant treatment of Merkel cell carcinoma. The results suggest that systemic therapy may improve outcomes in patients with high-risk disease, particularly those with nodal involvement. Furthermore, shorter chemotherapy regimens such as four cycles may provide sufficient therapeutic benefit while avoiding unnecessary toxicity.

Overall, our findings support a risk-adapted treatment approach in which adjuvant chemotherapy is considered for patients with stage III Merkel cell carcinoma and lymph node involvement. When systemic therapy is administered, four cycles may represent a reasonable treatment duration that balances potential survival benefit with treatment tolerability. However, given the retrospective nature of the study, these findings should be considered hypothesis-generating and warrant confirmation in prospective studies.

## 5. Conclusions

The present study evaluated the role of adjuvant chemotherapy in combination with radiotherapy in patients with resected Merkel cell carcinoma at high risk of recurrence, with the aim of determining whether systemic therapy is associated with improved outcomes compared with radiotherapy alone and whether the number of chemotherapy cycles influences survival. Our findings suggest that the addition of chemotherapy to adjuvant radiotherapy was associated with improved disease-free survival and overall survival, particularly in patients with advanced-stage disease and lymph node involvement. Notably, the four-cycle chemotherapy regimen was associated with the most favorable outcomes, whereas six cycles did not appear to provide additional survival benefit.

However, given the retrospective and non-randomized nature of the study, as well as potential imbalances between treatment groups and temporal heterogeneity across the study period, these findings should be interpreted with caution. The observed associations may be influenced by selection bias and residual confounding, despite multivariable adjustment. Overall, these results support the consideration of adjuvant chemotherapy in selected high-risk patients with Merkel cell carcinoma, particularly those with nodal involvement. Additionally, shorter chemotherapy regimens such as four cycles may represent a reasonable treatment approach that balances potential benefit with treatment-related toxicity. Nevertheless, these findings should be considered hypothesis-generating, and prospective studies are warranted to confirm the optimal role and duration of adjuvant chemotherapy in the modern era, particularly in the context of emerging immunotherapy strategies.

## Figures and Tables

**Table 1:** Patient characteristics by treatment group.

Characteristic	Overall (n = 104)	6 Cycles (n = 14)	4 Cycles (n = 63)	Radiotherapy Alone (n = 27)
**Age at diagnosis (years)**	70.6 ± 14.0	72.3 ± 11.7	68.1 ± 14.3	76.5 ± 13.6
**Sex**				
Male	61 (59%)	10 (71%)	39 (62%)	12 (44%)
Female	43 (41%)	4 (29%)	24 (38%)	15 (56%)
**Stage**				
IIB	12 (12%)	2 (14%)	4 (6%)	6 (22%)
IIIA	32 (31%)	3 (21%)	14 (22%)	15 (56%)
IIIB	60 (58%)	9 (64%)	45 (71%)	6 (22%)

Abbreviations: n, number.

**Table 2:** Survival outcomes by treatment group.

Outcome	Time Point	4 Cycles (n = 63) [95% CI]	6 Cycles (n = 14) [95% CI]	RT Only (n = 27) [95% CI]	*p*-Value (Log-Rank)
**DFS**	5 Years	65% [58–72%]	55% [43–67%]	40% [32–48%]	<0.001
	10 Years	52% [44–60%]	45% [32–58%]	28% [20–36%]	<0.001
	20 Years	45% [38–52%]	35% [21–49%]	12% [5–19%]	<0.0001
**OS**	5 Years	75% [68–82%]	60% [47–73%]	45% [34–56%]	<0.001
	10 Years	64% [56–72%]	50% [36–64%]	22% [14–30%]	<0.001
	20 Years	50% [43–57%]	30% [17–43%]	5% [1–9%]	<0.0001

Abbreviations: n, number; DFS, disease-free survival; OS, overall survival; CI, confidence interval; RT, radiotherapy.

## Data Availability

The data presented in this study are available on request from the corresponding authors.

## Abbreviations

The following abbreviations are used in this manuscript:

MCC	Merkel Cell Carcinoma
DFS	Disease-Free Survival
OS	Overall Survival
AJCC	American Joint Committee on Cancer
TNM	Tumor, Node, Metastasis
UV	Ultraviolet
MCPyV	Merkel Cell Polyomavirus
IMRT	Intensity-Modulated Radiotherapy
3D-CRT	Three-Dimensional Conformal Radiotherapy
GY	Gray
AUC	Area Under the Curve
CI	Confidence Interval
HR	Hazard Ratio
SD	Standard Deviation
